# Peripheral B-cell receptor immunoglobulin heavy chain repertoire as a potential biomarker for frequently relapsing nephrotic syndrome in children

**DOI:** 10.1080/0886022X.2026.2698338

**Published:** 2026-08-26

**Authors:** Anlu Feng, Xiaohan Li, Jin Cheng, Fengjun Guan

**Affiliations:** a Xuzhou Medical University, Xuzhou, Jiangsu, China; b The Affiliated Hospital of Xuzhou Medical University, Xuzhou, Jiangsu, China

**Keywords:** Frequent recurrence, primary nephrotic syndrome, B cell receptor repertoire, pediatric

## Abstract

**Background:**

This study aimed to investigate the distribution characteristics of the peripheral blood B cell receptor (BCR) repertoire immunoglobulin heavy chain (IGH) in children with frequently relapsing nephrotic syndrome (FRNS), explore its dynamic changes before and after glucocorticoid therapy, and evaluate its potential value as a biomarker for predicting FRNS.

**Methods:**

35 newly diagnosed children with primary nephrotic syndrome (PNS) admitted to Xuzhou Medical University Affiliated Hospital from September 2024 to September 2025 were divided into the FRNS group (*n* = 20) and the NFRNS group (*n* = 15), and 15 healthy children were selected as the control group. High-throughput sequencing was used to analyze the usage patterns, CDR-H3 length, somatic hypermutation (SHM), and diversity index of IGH genes in all subjects.

**Results:**

Before glucocorticoid treatment, compared with the NFRNS group, the FRNS group showed significantly increased IGH gene usage bias, shorter CDR-H3 length, higher SHM, and decreased BCR repertoire diversity (all *p* < 0.05). These abnormalities persisted or showed further deviation in the FRNS group after treatment but returned to near-normal levels in the NFRNS group. Pretreatment factors such as a lower Shannon index, a shorter CDR-H3 length, and a higher rate of SHM were significantly associated with poorer clinical remission in FRNS patients after treatment (all *p* < 0.01).

**Conclusion:**

Characteristics of the IGH repertoire in FRNS patients, especially the Shannon index, may serve as potential biomarkers for early risk stratification and personalized treatment of PNS in children.

## Introduction

Primary nephrotic syndrome (PNS) is one of the commonest glomerular diseases in children. Its hallmark is massive proteinuria, reflecting damage to the glomerular filtration barrier. Most cases are minimal change diseases. Exactly what triggers the condition is still unclear, but immune dysregulation is now thought to drive its onset, progression, and relapses [1].

Glucocorticoids (GC) remain the standard first-line treatment. They induce remission in 70–90% of children at initial presentation. Still, about half of those who respond will go on to develop frequently relapsing nephrotic syndrome (FRNS) [2]. These children need stronger immunosuppressants for longer periods, which brings higher risks – infections, growth delay – and may compromise long-term kidney function and quality of life [3,4]. Identifying immunological differences between children who will develop FRNS and those who will not is therefore critical for early risk stratification and personalized management [5].

Emerging evidence points to a pivotal role for B cells in PNS immunopathology [6,7]. Their numbers, subset composition, immunoglobulin output, and cytokine profiles all shift as the disease moves through different phases [8]. Clinically, the B-cell-depleting agent rituximab (RTX) significantly reduces the recurrence rate of FRNS, strongly suggesting that the behavior of B cells is related to disease activity [9,10]. But responses vary. Some children stay in remission long after B cells return; others relapse even when B cells are still profoundly depleted [11]. This heterogeneity suggests that relying solely on B cell counts is insufficient to monitor disease activity, and we need more sensitive and specific minimally invasive biomarkers.

The B cell receptor (BCR) is a core molecule that enables B cells to recognize antigens and initiate adaptive immune responses [12,13]. The BCR repertoire, including its diversity, clonal composition, CDR-H3 features, and somatic hypermutation (SHM) levels, comprehensively reflects the status of humoral immunity and clone amplification [14]. High-throughput sequencing-based BCR immunome analysis has been widely used to elucidate the pathogenesis, classification subtypes, and prognosis evaluation of various immune-related kidney diseases, such as membranous nephropathy and IgA nephropathy [15–17]. However, systematic research on the BCR library in pediatric PNS, particularly the direct comparison between FRNS and non-frequently relapsing nephrotic syndrome (NFRNS) and their evolution before and after GC treatment, is still limited [18].

In this study, we used high-throughput sequencing to analyze the diversity, clonal distribution, CDR-H3 features, and SHM levels of the peripheral blood IGH repertoire in newly diagnosed PNS children. We aimed to identify pretreatment immune repertoire differences between FRNS and NFRNS, track their evolution after GC treatment, and explore their association with short-term clinical response. The hope is that this might point to new biomarkers for early prognosis and, eventually, to more targeted treatment [19].

## Methods

### Samples

This study included 35 pediatric patients with newly diagnosed PNS who were hospitalized at the Affiliated Hospital of Xuzhou Medical University from September 2024 to September 2025. Based on the patients’ response to GC treatment and frequency of recurrence, they were divided into the FRNS group (*n* = 20) and the NFRNS group (*n* = 15) [20]. Additionally, 15 healthy children were recruited as a control group during the same period. PNS was diagnosed according to the 2016 Chinese guidelines for steroid-sensitive, relapsing/dependent nephrotic syndrome [2]. The three groups were comparable in gender (χ^2^ = 0.859, *p* = 0.593) and age (*F* = 0.069, *p* = 0.874). The study was approved by the hospital’s Medical Ethics Committee (XYFY2025-KL431-01), and written informed consent was obtained from all parents.

Exclusion criteria are as follows: a clear family history of kidney disease or hereditary nephropathy; secondary nephrotic syndrome (including lupus nephritis, purpuric nephritis, hepatitis B virus-associated nephritis, diabetic nephropathy, and kidney damage caused by other systemic diseases); concurrent autoimmune diseases, endocrine/metabolic disorders, malignancies, acute or chronic infections, or other severe systemic diseases; previous use of immunosuppressants that may affect immune function (such as GC, immunosuppressants, or biologics); steroid-resistant nephrotic syndrome; and incomplete clinical data or inability to cooperate with follow-up or specimen collection.

All pediatric patients with PNS received GC induction therapy immediately after diagnosis: prednisolone was administered orally in three divided doses at a dose of 2 mg/(kg d). After the urine protein turns negative, the medication is changed to once a day in the morning, continuing for 4 weeks. Subsequently, the patient enters the consolidation and maintenance phase, with prednisolone administered every other morning at a dose of 2 mg/(kg d), gradually reducing the dose after 4 weeks. The total treatment duration was 12 months [2].

Complete remission is defined as normalization of laboratory parameters and disappearance of clinical symptoms. Recurrence is defined as meeting any of the following criteria: a change from negative to (+++-++++) in urine protein in three consecutive morning urine samples; a urine protein/serum creatinine ratio of ≥2.0 mg/mg; or a 24-h urinary protein quantity (24 h UPQ) of ≥50 mg/kg [21]. NFRNS is defined as having ≤ 1 recurrence within 6 months or ≤3 recurrences within 1 year after initial complete remission, while FRNS is defined as having ≥2 recurrences within 6 months or ≥4 recurrences within 1 year after standard GC-induced remission. Clinical parameters, including 24 h UPQ, serum albumin, serum creatinine, and blood urea nitrogen, were collected before GC treatment and after 6 weeks of treatment. The 6-week post-treatment time point was selected based on the 2016 Chinese guidelines for steroid-sensitive, relapsing/dependent nephrotic syndrome in children, which recommend an initial induction phase of full-dose glucocorticoid therapy lasting 4–6 weeks [2]. By this time, clinical response (complete or partial remission) can be reliably assessed, and therapeutic decisions regarding the addition of immunosuppressive agents are typically made. Thus, the 6-week time point captures early treatment response and allows analysis of the association between early immune repertoire changes and short-term clinical outcomes.

### High-throughput sequencing of the IGH gene library

On the second day of admission and after 6 weeks of sufficient GC treatment, 4 mL of fasting peripheral venous blood was collected from all patients in the morning. Peripheral blood mononuclear cells (PBMCs) were separated by Ficoll density gradient centrifugation and treated with TRIzol^®^ reagent (Invitrogen, USA) lysed and stored at −80 °C. Total RNA was extracted using a total RNA extraction kit (Omega Bio tek, USA). Using 1 μg of high-quality total RNA as a template, six-base random primers and reverse transcriptase were used to synthesize cDNA first and double-stranded cDNA. The library fragments were enriched by PCR amplification, and the amplification products were electrophoretically purified by a gel extraction kit (Axgen, USA). The library was inspected by the Agilent 2100 Bioanalyzer. Finally, high-throughput sequencing was carried out on the Illumina NovaSeq 6000 platform in the paired-end, PE mode. The sequencing process is carried out by Shanghai Baipu Biotechnology Co., Ltd. [22].

### Analysis of key immunorepertoire metrics

A clonotype was defined as the combination of IGHV gene, IGHD gene, IGHJ gene, and CDR-H3 amino acid sequence. IGH gene alignment, assembly, and quantification were performed using MiXCR software (v4.3.2) against the IMGT reference database (release 2024-1). To minimize PCR duplicates and sequencing errors, clonotypes with a frequency below 0.01% of total productive IGH reads were discarded. In addition, an error-correction algorithm implemented in the software was applied to merge sequences with a single-nucleotide difference in the CDR-H3 region that shared the same V/J genes into the more abundant clonotype.

To correct for bias introduced by uneven sequencing depth, a rarefaction strategy was applied. The minimum number of productive IGH reads across all samples was identified (172,168 reads in the present study). For each sample, reads were randomly subsampled without replacement to this minimum count; this procedure was repeated 100 times, and the average values were used to reduce random sampling error. All diversity indices (Shannon index, Simpson index), CDR-H3 features, and SHM levels were calculated based on the rarefied data.

The diversity of the BCR repertoire is quantified by the Shannon index (H′) and Simpson index (D) [23]. The Shannon index reflects the richness and evenness of clones, with lower values indicating lower overall diversity. The Simpson index reflects the main clonal advantage, with lower values indicating significant clonal expansion and lower library diversity. The formula is as follows:

(1) $$D=1-\sum_{i=1}^{S}\;p_{i}^{2}$$

(2) $$H\prime =-\overset{S}{\underset{i=1}{\sum }}\;p_{i}\text{ln }\left(p_{i}\right)$$

where *S* is the total number of unique clonal types in the sample, and $p_{i}$ denotes the proportion of the *i*-th clonal type relative to the total valid BCR repertoire sequencing reads.

The SHM can evaluate the maturation of BCR affinity under antigen selection pressure. The higher the ratio, the stronger the antigen-driven clonal selection. The calculation method is the ratio of total point mutations to total nucleotides in the target area. The formula is as follows:

(3) $$\text{SHM rate}\left(\text{\%}\right)=\frac{\text{Total number of point mutations in IGH V gene region}}{\text{Total number of nucleotides in IGH V gene region}}\times 100\text{\%}$$

### Statistical analysis

Data are presented as mean ± SD for normally distributed continuous variables and as median for non-normally distributed ones. Intergroup comparisons were performed using an independent-samples *t*-test, Mann–Whitney U test or Kruskal–Wallis H test as appropriate. Categorical variables were analyzed with *χ*^2^ or Fisher’s exact test. Paired comparisons before and after treatment used a paired *t*-test, or Wilcoxon signed-rank test. Correlations between BCR repertoire features and clinical parameters were assessed by Spearman’s rank correlation. A two-tailed *p* < 0.05 was considered statistically significant. For intergroup comparisons of differentially expressed IGH genes, the false discovery rate (FDR) was controlled using the Benjamini–Hochberg method, and an FDR < 0.05 was considered statistically significant. All analyses were conducted using SPSS 26.0 and R 4.5.2 [24].

## Results

### General clinical characteristics of the pediatric patients

**Figure 1.**
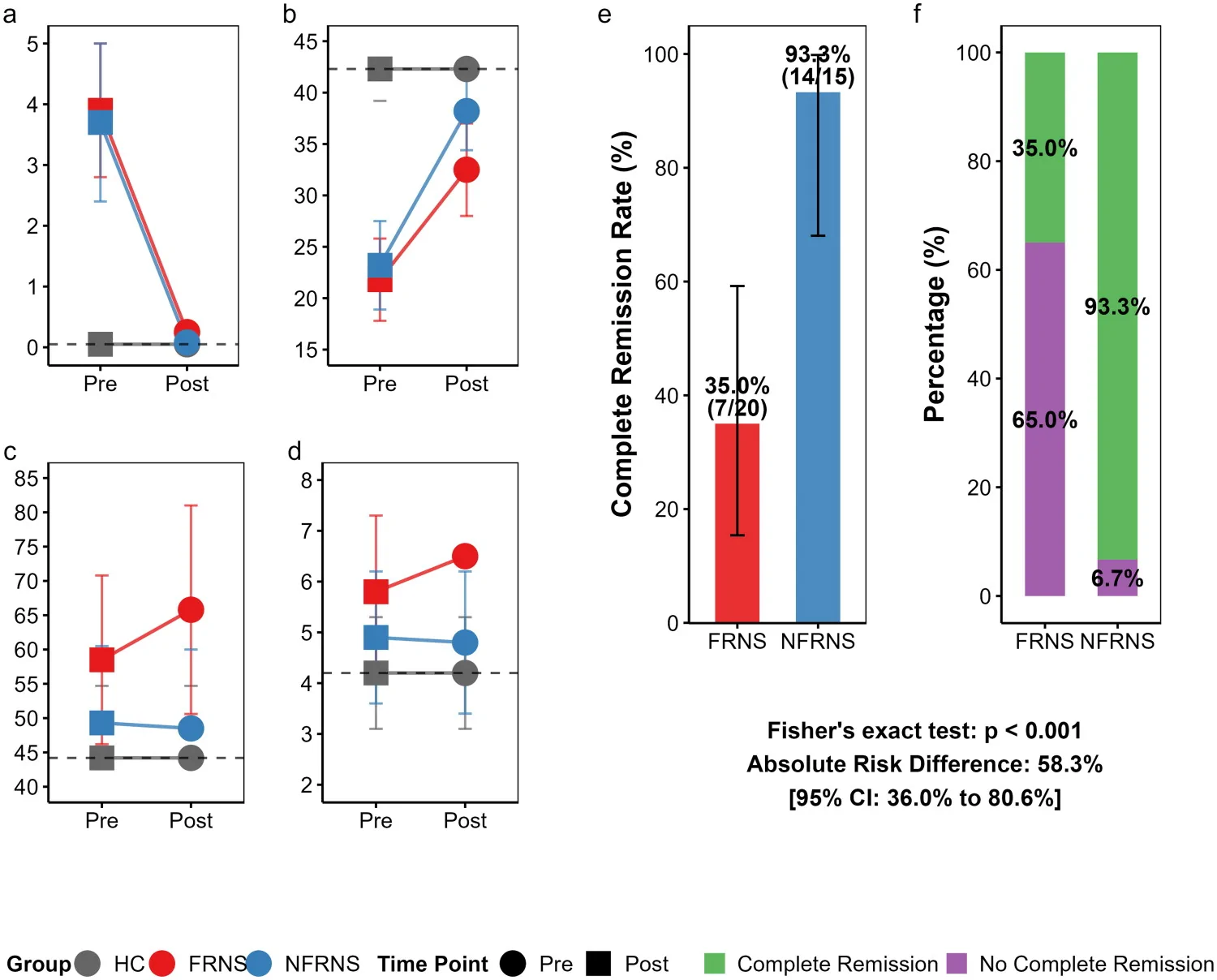
Dynamic changes in clinical parameters and complete response rate of children with nephrotic syndrome before and after glucocorticoid treatment (a–d) Line graph of (a) 24-h urinary protein, (b) serum albumin, (c) serum creatinine, (d) blood urea, HC, FRNS, NFRNS before and 6 weeks after treatment. (e) The CR rates of FRNS and NFRNS (n/N: FRNS 35.0% [7/20], NFRNS 93.3% [14/15]). (f) Stacked bar chart for alleviating states. Fisher’s exact test, *p* < 0.001. The absolute risk difference is 58.3% (95%CI: 36.0–80.6%).

Before GC treatment, the 24 h UPQ and serum albumin levels in the FRNS and NFRNS groups were significantly lower than those in the HC group (both *p* < 0.05), with no significant difference between the groups. There was no significant difference in serum creatinine and blood urea among the three groups (*p* > 0.05). All patients were sensitive to GC, but the treatment response varied at 6 weeks (Table 1, Figure 1). More than 90% of NFRNS patients achieved complete remission, with 24-h UPQ (0.08 ± 0.05 g/24 h) and serum albumin (38.2 ± 3.8g/L) returning to normal, and serum creatinine and blood urea within normal ranges. In contrast, only 30–40% of FRNS patients achieved complete remission, with their 24-h UPQ (0.25 ± 0.15g/24h) and serum albumin (32.5 ± 4.5 g/L) significantly lower than NFRNS, and serum creatinine and blood urea significantly increased (*p* < 0.01), suggesting more severe kidney damage [25].

**Table 1.** Clinical characteristics of participants Pre- and 6 weeks Post-glucocorticoid therapy.

Group	n	24h urine protein (g/24h)	Serum albumin (g/L)	Serum creatinine (μmol/L)	Blood urea (mmol/L)
HC	15	0.05 ± 0.02	42.3 ± 3.1	44.2 ± 10.5	4.2 ± 1.1
FRNS (Pre)	20	3.9 ± 1.1***	21.8 ± 4.0***	58.5 ± 12.3^††^	5.8 ± 1.5
FRNS (Post)	20	0.25 ± 0.15***	32.5 ± 4.5***	65.8 ± 15.2	6.5 ± 1.8
NFRNS (Pre)	15	3.7 ± 1.3***	23.2 ± 4.3***	49.3 ± 11.2^††^	4.9 ± 1.3
NFRNS (Post)	15	0.08 ± 0.05***	38.2 ± 3.8***	48.5 ± 11.5	4.8 ± 1.4

^a^ Data are expressed as mean ± standard deviation (SD).

^b^ FRNS, frequently relapsing nephrotic syndrome; HC, healthy control; NFRNS, non-frequently relapsing nephrotic syndrome; Post, 6 weeks post-glucocorticoid therapy; Pre, pretreatment.

^c^ ***p < 0.001 for intragroup pre- vs post-treatment comparison (FRNS/NFRNS).

^d^ ††*p* = 0.029 for FRNS (Pre) vs NFRNS (Pre).

### IGH gene lineage and expression characteristics

**Figure 2.**
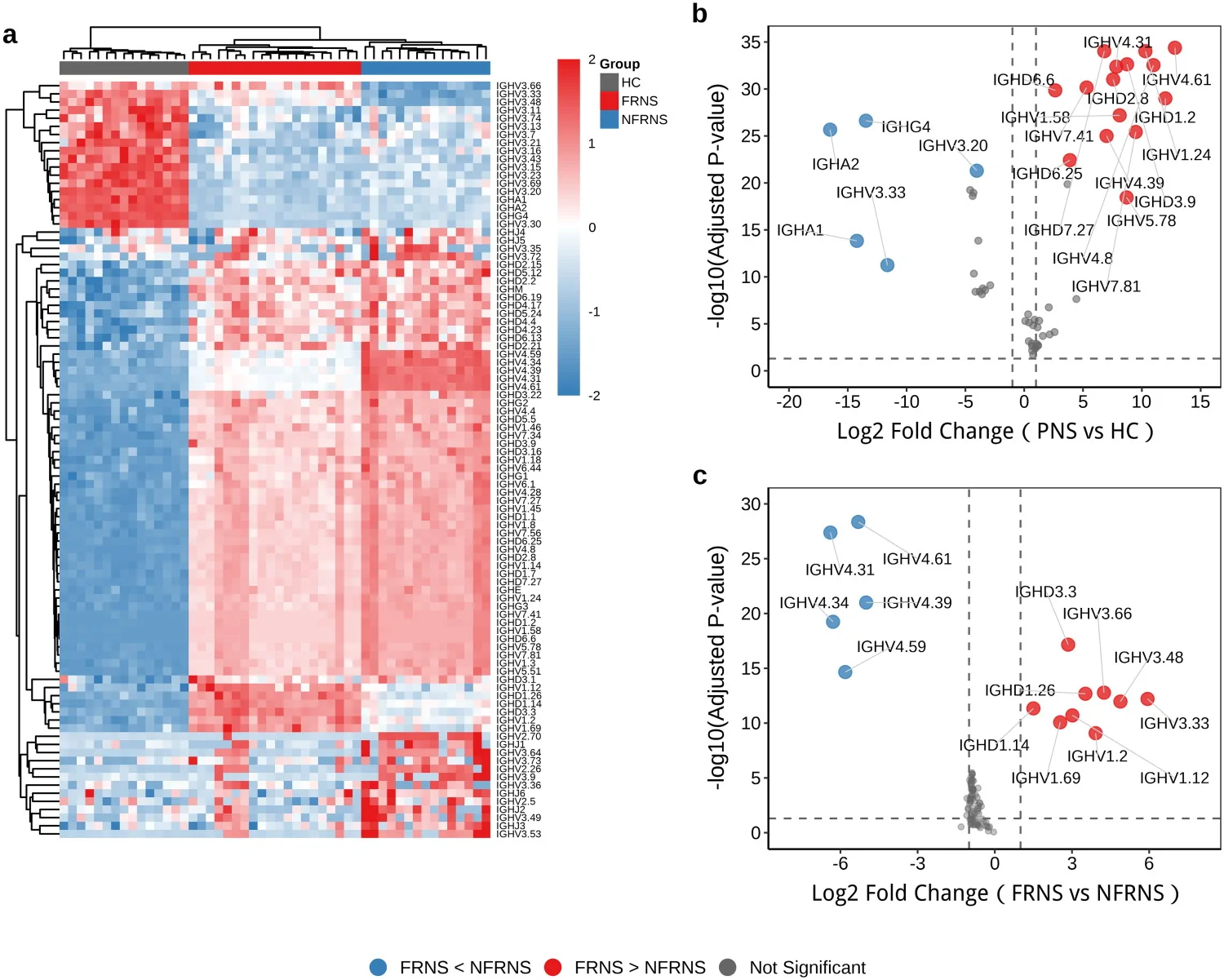
IGH gene expression patterns and differential expression analysis. (a) Heatmap of row-wise *Z*-score normalized IGH gene expression in HC, FRNS, NFRNS groups. (b) Volcano plot (HC vs PNS): *x*-axis log_2_ fold change (PNS/HC), *y*-axis − log_10_ (adjusted *p*-values calculated using the Benjamini–Hochberg FDR method); key dysregulated genes annotated. (c) Volcano plot (FRNS vs NFRNS): *x*-axis log_2_ fold change (FRNS/NFRNS), *y*-axis − log_10_ (adjusted *p*-values calculated using the Benjamini–Hochberg FDR method); top dysregulated genes are annotated.

We performed high-throughput sequencing of IGH genes on 50 samples from children with HC, FRNS, and NFRNS and identified a total of 86 IGHV, 25 IGHD, and 10 IGHC genes. Compared with the HC group, pediatric PNS patients, including FRNS and NFRNS, showed changes in the frequency of IGH gene usage. The differential expression heatmap (Figure 2) shows 77 differentially expressed IGH transcripts between pediatric PNS patients and HC, with 59 upregulated and 18 downregulated. There are also different IGH gene expression profiles between the FRNS group and the NFRNS group, with nine genes upregulated and five genes downregulated in the FRNS group. It is worth noting that the upregulated IGHV3-33, IGHV3-48, and IGHV3-66 genes in the FRNS group were downregulated throughout pediatric PNS patients, while the downregulated IGHV4-31, IGHV4-34, IGHV4-49, IGHV4-59, and IGHV7-61 genes were upregulated in pediatric PNS patients. These differences persisted before and after GC treatment, and no intragroup expression changes were observed.

### CDR-H3 length and distribution characteristics

**Figure 3.**
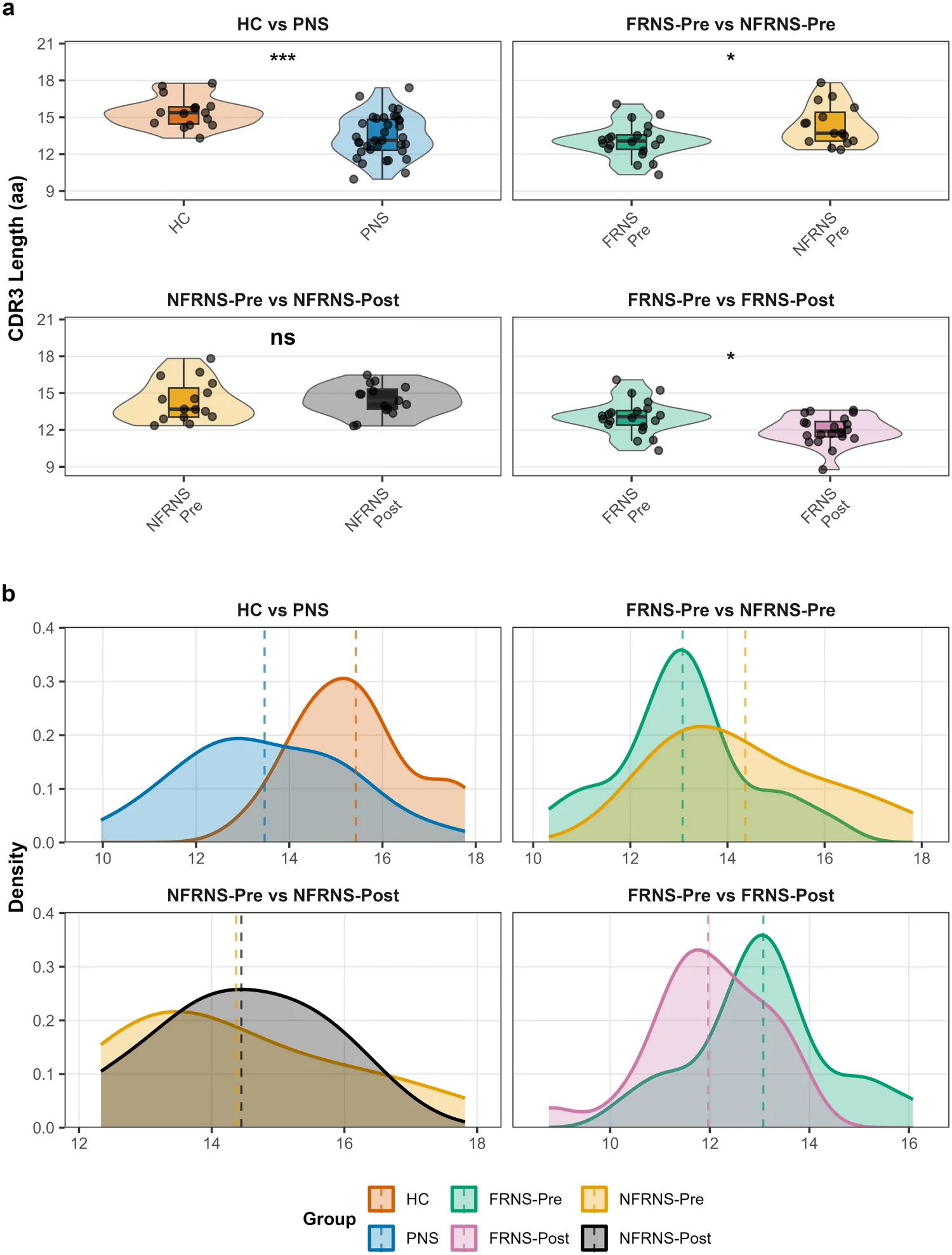
CDR-H3 length distribution across groups: (a) box plots overlaid with violin plots and jittered points for CDR-H3 length in four comparisons: HC vs PNS, FRNS-Pre vs NFRNS-Pre, NFRNS-Pre vs NFRNS-Post, FRNS-Pre vs FRNS-Post. Unpaired t-test, ***p < 0.001. (b) Density curves for the same comparisons.

The length and distribution of CDR-H3 showed significant intergroup differences (*p* < 0.001) (Table 2, Figure 3). The average length of CDR-H3 in the HC group was 15.2 ± 1.5 aa, with a normal distribution (peak at 14–16.5 aa), while CDR-H3 in pediatric PNS patients was shortened (mean 13.5 ± 1.8 aa, *p* < 0.001), with a narrow distribution and leftward shift (peak at 12–14 aa). Before treatment, the CDR-H3 of the FRNS group was significantly shorter than that of NFRNS (12.8 ± 1.6 vs 14.1 ± 1.7 aa, *p* < 0.01), with a narrower distribution and a significant left shift. After 6 weeks of GC treatment, CDR-H3 in the FRNS group further shortened (mean 12.0 ± 1.4 aa, *p* < 0.01), and its distribution continued to shift to the left without improvement, while NFRNS returned to near HC levels (15.0 ± 1.6 aa), with a wider distribution and a return to normal distribution.

**Table 2.** BCR repertoire characteristics before and after glucocorticoid therapy.

Group	*n*	CDR-H3 length (aa)		SHM (%)		Shannon index		Simpson index	
		Mean ± SD	Median [IQR]	Mean ± SD	Median [IQR]	Mean ± SD	Median [IQR]	Mean ± SD	Median [IQR]
HC	15	15.2 ± 1.5	15.0 [14.0–16.5]	8.0 ± 3.0	8.0 [6.0–10.0]	8.5 ± 0.8	8.5 [7.8–9.2]	0.92 ± 0.04	0.92 [0.89–0.95]
PNS (Pre)	35	13.5 ± 1.8***	13 5 [12.0–15.0]	15.0 ± 5.0**	15.0 [11.0–19.0]	6.2 ± 1.2***	6.2 [5.3–7.1]	0.78 ± 0.08***	0.78 [0.72–0.84]
FRNS (Pre)	20	12.8 ± 1.6^***,††^	12.8 [11.5–14.0]	18.0 ± 4.0^***,†^	18.0 [15.0–21.0]	5.5 ± 1.1^***,††^	5.5 [4.7–6.3]	0.72 ± 0.07^***,††^	0.72 [0.67–0.77]
FRNS (Post)	20	12.0 ± 1.4^***,‡‡^	12.0 [10.8–13.2]	20.0 ± 5.0^***,‡^	20.0 [16.0–23.0]	5.0 ± 1.0^***,‡‡^	5.0 [4.2–5.8]	0.68 ± 0.08^***,‡‡^	0 68 [0.62–0.74]
NFRNS (Pre)	15	14.1 ± 1.7*	14.1 [12.8–15.3]	12.0 ± 4.0*	12.0 [9.0–15.0]	6.8 ± 1.0*	6.8 [6.0–7.6]	0.81 ± 0.06*	0.81 [0.77–0.85]
NFRNS (Post)	15	15.0 ± 1.6^ⁿˢ^	15.0 [13.7–16.2]	110 ± 40^ⁿˢ^	11.0 [8.0–14.0]	7.2 ± 0.9^ⁿˢ^	7.2 [6.5–7.9]	0.86 ± 0.05^ⁿˢ^	0.86 [0.83–0.89]

^a^ Data expressed as mean ± standard deviation (SD) and median [interquartile range (IQR)]; SHM shown as a percentage.

^b^ FRNS, frequently relapsing nephrotic syndrome; HC, healthy control; NFRNS, non-frequently relapsing nephrotic syndrome; PNS, primary nephrotic syndrome, Post, 6 weeks post-glucocorticoid therapy; Pre, pretreatment.

^c^ *p < 0.05, **p < 0.01, ***p < 0.001 vs HC group; ^ns^, not significant vs HC group.

^d †^*p* < 0.05, ^††^*p* < 0.01: FRNS (Pre) vs NFRNS (Pre).

^e ‡^*p* < 0.05, ^‡‡^*p* < 0.01: FRNS (Post) vs FRNS (Pre).

### SHMs and BCR repertoire diversity indices

**Figure 4.**
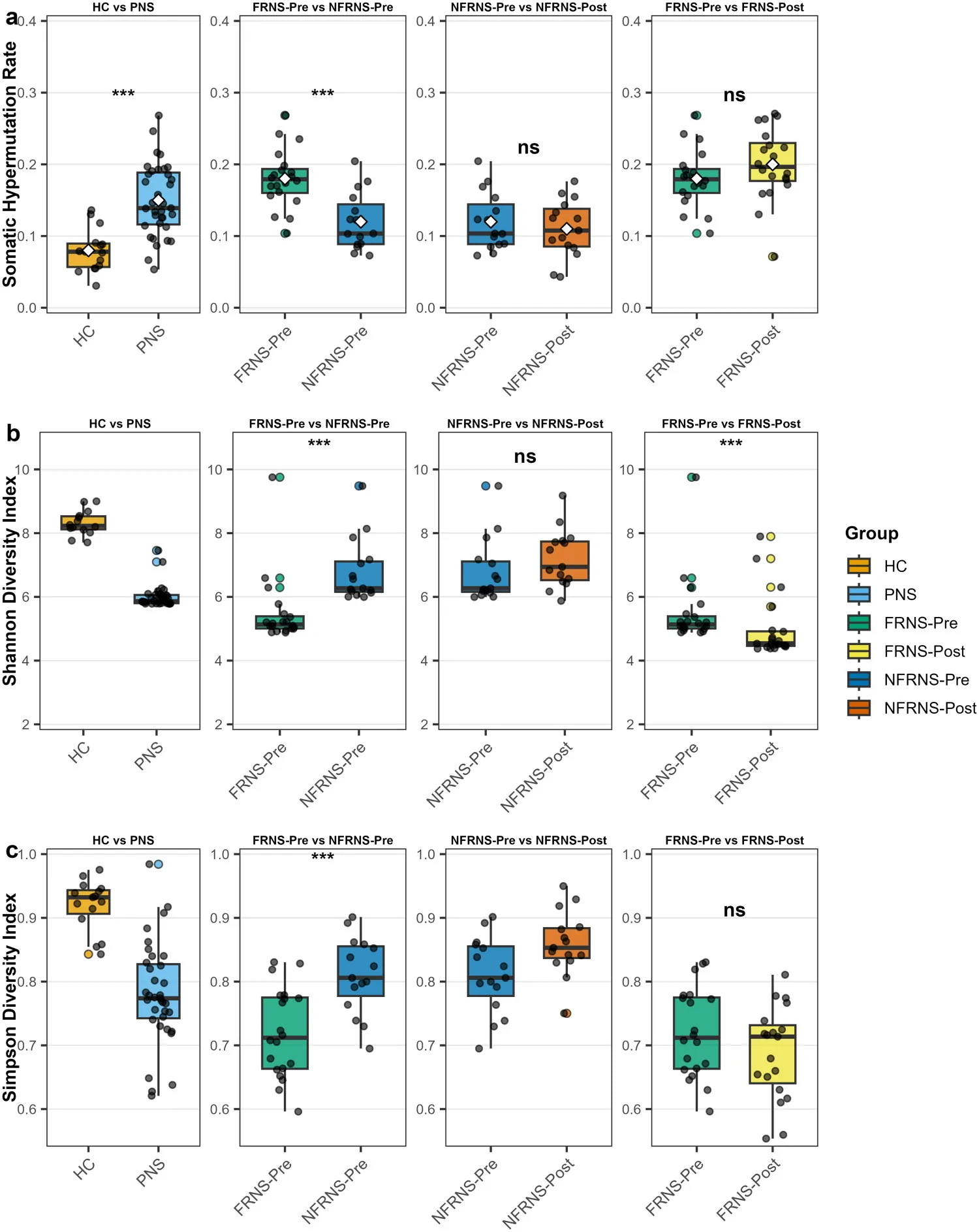
SHM and BCR repertoire diversity indices across groups. Box plots (overlaid with jittered points) for (a) SHM, (b) Shannon index (H′), (c) Simpson index in four comparisons: HC vs PNS, FRNS-Pre vs NFRNS-Pre, NFRNS-Pre vs NFRNS-Post, FRNS-Pre vs FRNS-Post. Wilcoxon rank-sum test: *p < 0.05, **p < 0.01, ***p < 0.001.

There were significant differences in SHM among the groups (Table 2, Figure 4). The SHM in the HC group was 0.08 ± 0.03, while it was significantly increased in the PNS group (0.15 ± 0.05, *p* < 0.01). Before treatment, the SHM in the FRNS group was higher than that in the NFRNS group (0.18 ± 0.04 vs 0.12 ± 0.04, *p* < 0.05). After 6 weeks of GC treatment, the SHM in the NFRNS group decreased to near normal levels (0.11 ± 0.04), while the FRNS group continued to increase (0.20 ± 0.05), and the difference between the groups widened (*p* < 0.01).

Compared with HC, the BCR spectrum diversity (H’, D) of pediatric PNS patients was significantly reduced (H′: 6.2 ± 1.2 vs 8.5 ± 0.8, D: 0.78 ± 0.08 vs 0.92 ± 0.04, both *p* < 0.001). Before treatment, the H′ (5.5 ± 1.1) and D (0.72 ± 0.07) of the FRNS group were lower than those of NFRNS (6.8 ± 1.0, 0.81 ± 0.06, respectively), *p* < 0.01). After treatment, the H′ and D parts of the NFRNS group recovered, while the FRNS group further decreased.

### BCR repertoire features and clinical outcome correlations

**Figure 5.**
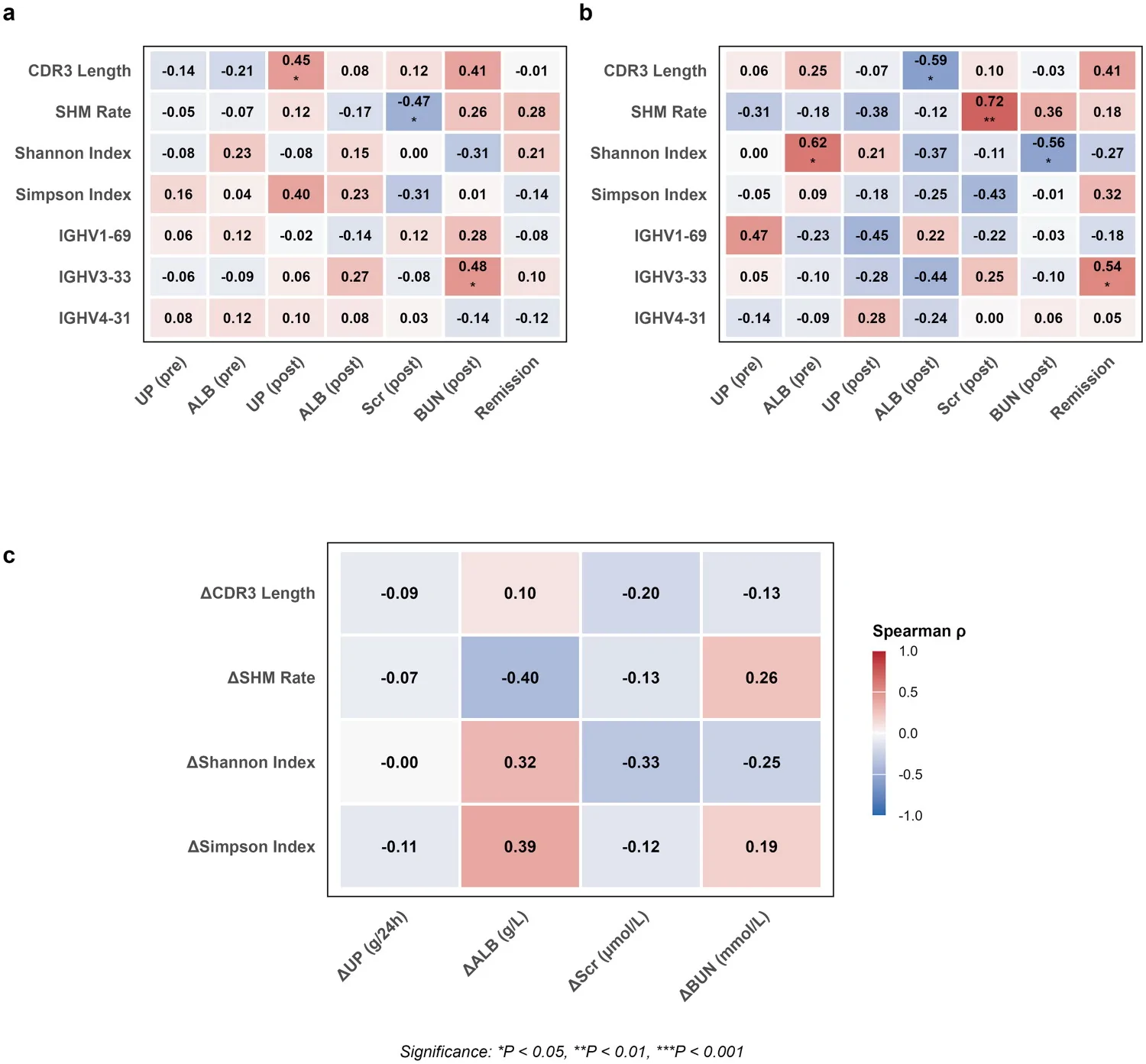
Correlations of BCR repertoire features with clinical parameters. Heatmaps of Spearman’s correlation coefficients (*ρ*) for (a) FRNS and (b) NFRNS, pretreatment immune parameters vs clinical parameters. (c) FRNS, Δimmune parameters vs Δclinical parameters.

Spearman rank correlation analysis identified a specific correlation between BCR library features and clinical outcomes in FRNS, while this significant correlation was not observed in the NFRNS group (all *p* > 0.05, Figure 5). In the FRNS group, there was a strong negative correlation between pretreatment H′ and 24-h UPQ (*ρ* = −0.72, *p* < 0.01) and non-remission state (*ρ* = −0.81, *p* < 0.001) after treatment. The SHM before treatment was positively correlated with the UPQ 24 h before treatment (*ρ* = 0.65, *p* < 0.01). The length of CDR-H3 before treatment was positively correlated with serum albumin after treatment (Φ = 0.58, *p* < 0.05). The expression of IGHV1-69 was correlated with UPQ at 24 h after treatment (*p* = 0.51, *p* < 0.05), while IGHV4-31 was correlated with clinical remission (*p* = −0.56, *p* < 0.05).

**Figure 6.**
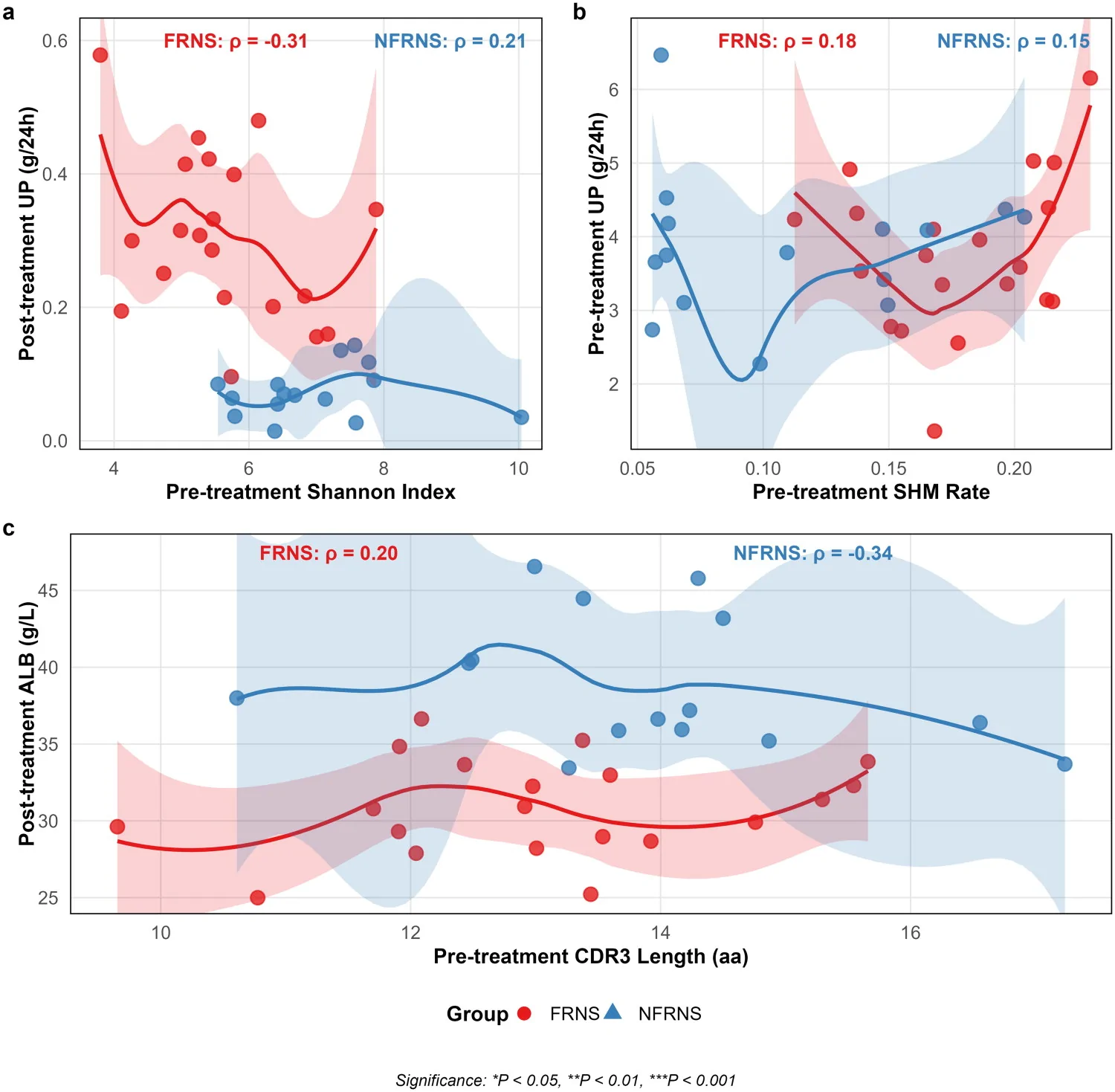
Associations of key immune parameters with clinical outcomes: (a) pretreatment H′ vs post-treatment UP; (b) pretreatment SHM vs pretreatment UP; (c) pretreatment CDR-H3 length vs post-treatment ALB. Spearman’s *ρ* and significance shown per group.

The dynamic analysis of FRNS showed that Δ H’ was negatively correlated with Δ 24 h UPQ (*ρ* = −0.69, *p* < 0.01) and positively correlated with Δ serum albumin (*ρ* = 0.58, *p* < 0.05) (Figure 6). The length of Δ CDR-H3 (*ρ* = −0.45, *p* < 0.05) and Δ *D* (*ρ* = −0.51, *p* < 0.05) are negatively correlated with Δ 24 h UPQ. The Δ SHM showed no correlation (*p* = 0.12, *p* > 0.05). There was no correlation between Δ immune indicators and Δ serum creatinine or Δ blood urea (both *p* > 0.05).

### ROC curve analysis

**Figure 7.**
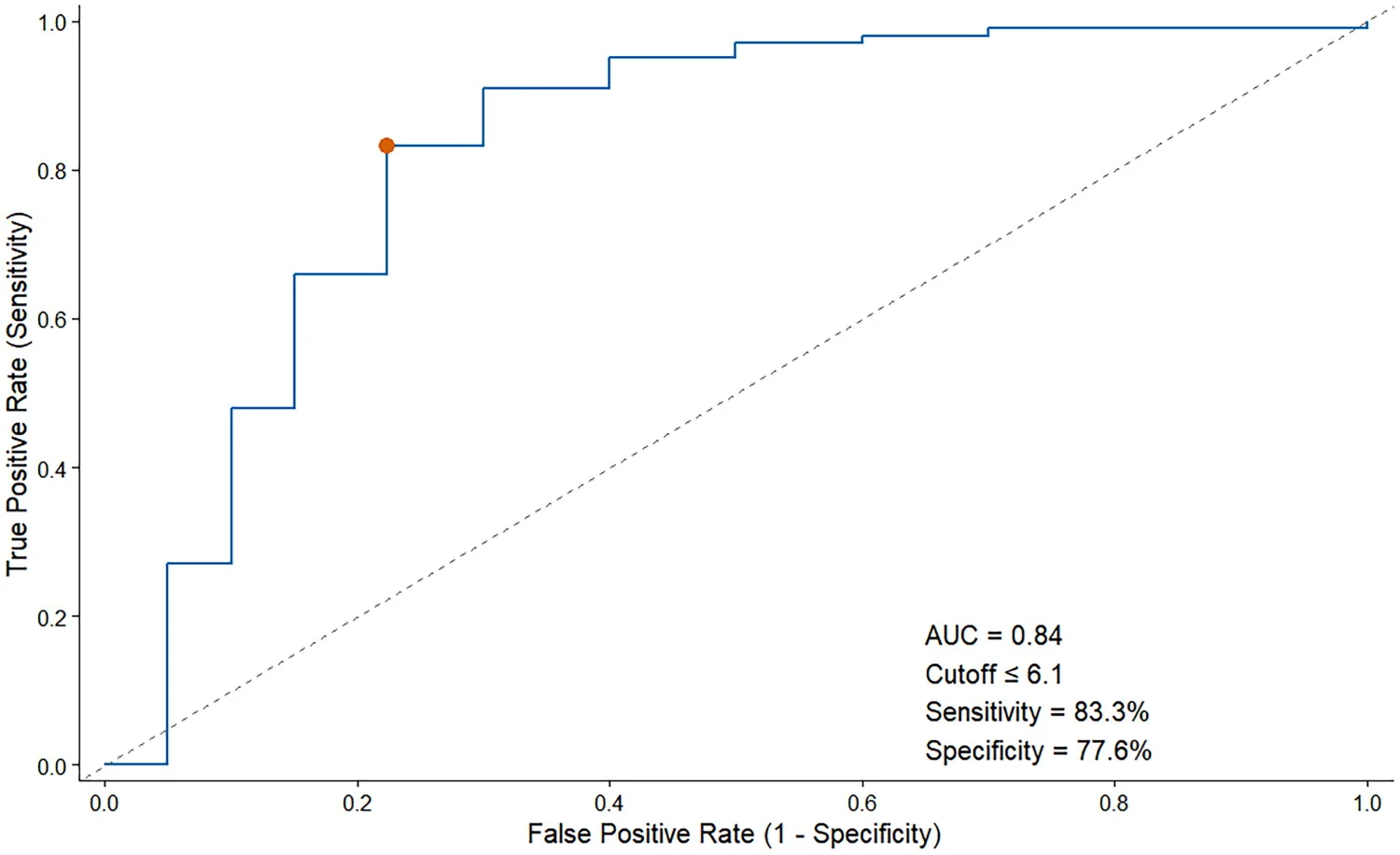
ROC curve of pretreatment Shannon index for discriminating FRNS from NFRNS. The area under the curve (AUC) was 0.84 (95% CI: 0.72–0.96). The optimal cutoff value was a Shannon index ≤6.1, yielding a sensitivity of 83.3% and a specificity of 77.6%.

Using the pretreatment Shannon index as the test variable and the classification of FRNS versus NFRNS as the state variable, ROC curve analysis demonstrated that the Shannon index discriminated FRNS from NFRNS with good accuracy. The area under the curve (AUC) was 0.84 (95% CI: 0.72–0.96, *p* < 0.001). The optimal cutoff value determined by the Youden index was ≤6.1, yielding a sensitivity of 83.3% (95% CI: 67.2–93.6%) and a specificity of 77.6% (95% CI: 64.7–87.5%) (Figure 7).

## Discussion

This study systematically describes the characteristics of the peripheral blood BCR IGH immune repertoire in children with FRNS. There are three main findings. Firstly, before treatment, the FRNS group showed significantly different immune repertoire characteristics compared to the HC and NFRNS groups, manifested as lower diversity, shorter CDR-H3 length, higher SHM, and bias in the use of IGHV genes. Secondly, these abnormalities in the FRNS group not only did not improve after 6 weeks of GC treatment but instead showed further deviation, whereas most indicators in the NFRNS group returned to near-normal levels. The third is the Shannon index before treatment, which is closely related to the short-term clinical outcomes of FRNS, suggesting that it can serve as an early prognostic biomarker [26].

The continuous decrease in BCR repertoire diversity (i.e., Shannon index and Simpson index) in children with FRNS suggests that, under sustained antigen stimulation or abnormal autoimmune responses, the immune system may undergo pathological oligoclonal amplification, which in turn may lead to impaired generation and recruitment of new lymphocytes and reduced immune response capacity [27], suggesting an immune exhaustion state [13]. In addition, the CDR-H3 region in children with FRNS is significantly shortened, and the abnormal shortening of the CDR-H3 region, which serves as the core functional area for BCR recognition of antigens [28], is often considered a typical sign of insufficient B cell bank regeneration ability, immune function decline, or immune aging [29]. It is worth noting that GC treatment did not reverse the depletion trend of FRNS but restored diversity in the NFRNS group, highlighting the essential immunological differences between the two clinical phenotypes.

The sustained increase in SHM before and after treatment in children with FRNS suggests the existence of a sustained antigen-driven affinity maturation process [30]. SHM usually occurs in the germinal center, relies on T cell assistance, and is a marker of B cells undergoing antigen stimulation [31,32]. The continued increase in SHM after hormone therapy suggests the possible presence of long-lived memory B cells or plasma cells that are relatively insensitive to steroid-induced apoptosis and that may remain in an activated state for prolonged periods [33]. Under nonspecific stimuli such as infection or fatigue, these cells could be reactivated, may secrete substantial amounts of pathogenic antibodies, and might disrupt the glomerular filtration barrier, thereby contributing to disease recurrence [34]. This explanation is consistent with the clinical efficacy of RTX, which can achieve deep intervention in the pathogenesis of diseases by specifically clearing CD20 + B cells, including the aforementioned memory B cells with pathogenic potential [11,35]. Therefore, FRNS patients who are insensitive to GC therapy that can only inhibit B cell activation and induce transient apoptosis can still benefit from RTX therapy [36,37].

FRNS patients exhibit significant specificity in the frequency of IGHV gene usage, with differentially expressed genes such as IGHV1-69 and IGHV4-31 closely associated with treatment response. This IGHV gene usage bias is unlikely to be random and may instead result from preferential amplification of B cell clones under specific antigen selection pressure. Although previous studies have suggested that these IGHV genes might encode pathogenic antibody molecules [38], it is important to emphasize that our differential expression analysis alone does not directly demonstrate pathogenicity. The characteristic IGHV gene profile described here provides research clues for exploring the molecular etiology of FRNS, but these findings are hypothesis-generating. The antigen specificity and pathogenic relevance of the encoded antibodies require further validation through additional experiments, e.g. single-cell sequencing, recombinant antibody expression, and antigen-binding assays [39].

From a clinical translation perspective, the pretreatment Shannon index shows potential as a predictor of adverse treatment response. It is negatively correlated with post-treatment 24-h urinary protein levels and clinical remission status, and the dynamic changes in the Shannon index before and after treatment parallel the degree of clinical remission. ROC curve analysis further supported the discriminative ability of the pretreatment Shannon index for FRNS (AUC = 0.84; at a cutoff of 6.1, the sensitivity and specificity were 83.3% and 77.6%, respectively). These findings suggest that the Shannon index may serve as a potential auxiliary indicator for distinguishing FRNS from NFRNS, which may aid early risk stratification in children with newly diagnosed PNS [40]. Thus, in children with newly diagnosed PNS, measuring BCR immune repertoire characteristics before treatment may help identify those at high risk of FRNS, providing a reference for individualized monitoring and treatment planning in clinical practice [41].

This study has several limitations. Firstly, as a single-center study with limited sample size, extrapolation of results should be cautious. The follow-up period only covers short-term treatment response and fails to evaluate the predictive value of immune markers for long-term recurrence and renal function prognosis. Furthermore, glucocorticoid-induced immune reconstitution may require longer periods (e.g., beyond 6 months), and the present study only captured repertoire changes within 6 weeks of therapy, precluding assessment of longer-term immune recovery and its relationship with sustained relapse risk. In addition, the FRNS group had slightly higher baseline 24-h urinary protein and lower serum albumin levels compared with the NFRNS group (Table 1). This incomplete balance in baseline disease severity may have contributed to some of the observed differences in BCR repertoire features between the two groups. Because of the limited sample size, we did not perform multivariable regression analysis to adjust for potential confounding by baseline clinical parameters. Therefore, we cannot completely rule out the possibility that the BCR repertoire abnormalities observed in FRNS patients partly reflect more severe baseline disease rather than intrinsic immune alterations specifically associated with the frequently relapsing phenotype. Future studies with larger cohorts are needed to further validate the independent association between BCR repertoire characteristics and FRNS using multivariable analysis or propensity score matching. Secondly, the research focuses on the BCR immune repertoire without synchronously analyzing the TCR repertoire and cytokine profile [42,43]. The interaction between T cells and B cells in FRNS requires further investigation. As for the IGHV genes associated with this disease that we discovered, their pathogenic potential remains suggestive rather than proven. Thus, the differential IGHV gene expression observed in this study should be regarded as exploratory findings rather than evidence of pathogenicity. Whether they truly drive this process and the antigens they may target, further work such as single-cell sequencing [44], recombinant antibody expression, and binding detection should help clarify the issue. Despite these limitations, this study provides the first comprehensive landscape of BCR IGH repertoire alterations in FRNS and identifies the Shannon index as a promising candidate biomarker for early risk stratification, warranting further validation in larger, multi-center cohorts.

Taken together, we observed a set of persistent BCR repertoire abnormalities in children with FRNS – lower diversity, shorter CDR-H3, higher SHM, and biased IGHV usage – that were already present before treatment. These features correlated strongly with short-term clinical response, and among them the Shannon index stood out as the most promising early indicator. Our findings shed light on the immunopathology of FRNS and provide initial evidence that repertoire analysis could help guide risk stratification and individualized management in pediatric PNS.

## Conclusion

Children with FRNS exhibit characteristic changes in the BCR immune repertoire prior to treatment, including decreased diversity, shortened CDR-H3, increased SHM, and biased use of IGHV genes. These abnormalities persist after hormone therapy and are associated with poor short-term treatment response. Among them, the Shannon index may serve as an auxiliary indicator for distinguishing FRNS from NFRNS and guiding early risk stratification. This study provides new ideas for the immunological typing and individualized management of PNS in children.

## Data Availability

The datasets generated and/or analyzed during the current study are available from the corresponding author on reasonable request. All clinical and analytical data are included within the manuscript and its supplementary materials.
